# Intravenous versus Intra-Arterial Thrombolysis in Ischemic Stroke: A Systematic Review and Meta-Analysis

**DOI:** 10.1371/journal.pone.0116120

**Published:** 2015-01-08

**Authors:** Qing-feng Ma, Chang-biao Chu, Hai-qing Song

**Affiliations:** Department of Neurology, Xuan Wu Hospital, Capital Medical University, Beijing, China; University of Münster, GERMANY

## Abstract

**Background:**

Reperfusion following ischemic stroke can be attained by either intravenous thrombolysis (IVT) or intra-arterial thrombolysis (IAT). Only a limited number of randomized prospective studies have compared the efficacy and safety of IVT and IAT. This meta-analysis investigated possible clinical benefits of IAT relative to IVT in patients with acute ischemic stroke.

**Methods:**

We searched the PubMed, Cochrane, and Google Scholar databases through October 2013 for manuscripts that describe the findings of randomized controlled or prospective studies that evaluated the outcomes of patients with ischemic stroke who were treated with IVT or IAT. The clinical outcome measures were score on the modified Rankin scale (mRS) and mortality at 90 days. A favorable outcome was defined as an mRS score of 0 to 2.

**Results:**

For the mRS, the combined odds ratio (OR) of 3.28 (95% confidence interval (CI), 1.91 to 5.65, P < 0.001) indicated that patients who received IAT had a significantly higher chance for a favorable outcome than did those who received IVT. For mortality, the OR indicated that IAT therapy significantly reduced the proportion of patients who died within 90 days of the procedure (combined OR, 0.40; 95%CI, 0.17 to 0.92; P = 0.032).

**Conclusion:**

This meta-analysis determined that IAT conferred a significantly greater probability of achieving a favorable outcome compared with IVT. There was also a significant difference in mortality rates between IAT and IVT. The studies included in this analysis were small and heterogeneous; therefore, larger randomized prospective clinical studies are necessary to further investigate this issue.

## Introduction

Treatment of acute ischemic stroke attempts to open the occluded blood vessels in order to re-establish blood flow and to improve outcomes [1]. Reperfusion can be attained by intravenous thrombolysis (IVT) or by intra-arterial thrombolysis (IAT) [1]. It is recommended that IVT be given as first-line therapy for acute ischemic stroke within 4.5 hours of the onset of symptoms; however, approximately 50% of patients treated with IVT do not recover and die [2, 3]. In addition, although overall recanalization rates are approximately 46%, those of IVT are low when the occlusion is in a large artery. In these cases, published rates range from 4% to 68% and depend upon both the location of the occlusion and the study [4–11].

The findings from a number of studies suggest that IAT may be a reasonable alternative to IVT. In some studies, IAT is associated with higher rates of recanalization than is IVT [12–14]. There are several potential advantages to IAT, such as angiographic planning to customize therapy, locoregional injection, and the ability to use mechanical devices to speed up the recanalization rate [15]. There is a delay in treatment with IAT relative to IVT, and this delay may lessen the advantages of the procedure, since time to treatment is a major predictor of outcome for acute stroke [16, 17]. As is the case with IVT, one of the risks of IAT is intracranial hemorrhage; another is the risk of procedural complications such anaphylaxis and systemic bleeding. In one of the earliest clinical trials of IAT [8], the rates of all intracranial hemorrhages (ICH) at 24 hours and mortality at 90 days were approximately 42% and 27%, respectively. Later trials and studies reported mortality rates of 7% to 29% [18, 19], and rates of hemorrhage that ranged from 0% to 33% [19, 20]. In a study of patients who received IAT (together with other endovascular treatments) after ≥8 hours, Natarajan and colleagues documented approximate rates of 33% for both mortality at 90 days and immediate hemorrhage (subarachnoid and intracranial) [21]. The risk of ICH with IAT may be associated not only with the dose of thrombolytic used during IAT [22], but also with the severity of the stroke [23]. Regarding other complications, the PROACT II trial reported that 1% of patients receiving IAT with recombinant prourokinase experienced anaphylaxis and 7% experienced systemic hemorrhage [13]. Only a limited number of prospective randomized studies have compared the efficacy and safety of IVT and IAT [13, 18, 20, 24–26], and many of these were small or did not use the same thrombolytic agent with each mode of administration. Several of the studies found that there were no significant differences between treatments in the proportion of patients who were alive without disability, had symptomatic hemorrhage, or died [24–26]. Others found that IAT showed a clinical benefit compared with IVT for several of these outcomes [13, 18, 20]. To gain further insight into possible clinical benefits of IAT relative to IVT, we performed a meta-analysis to evaluate the efficacy and safety of IAT and IVT in patients with acute ischemic stroke.

## Methods

### Search strategy

Meta-analyses do not involve human subjects and do not require IRB review. We searched the PubMed, Cochrane, and Google Scholar databases through October 2013 for manuscripts that describe the findings of randomized controlled or prospective studies and that evaluated outcomes in patients with ischemic stroke who were treated with IVT or IAT. The following search terms were used: stroke, brain ischemia, ischemic stroke, streptokinase, urokinase, thrombolytic therapy, tissue plasminogen activator, intra-arterial, intravenous, and thrombolysis. A study was excluded if it did not report relevant treatment outcomes, if it was a single arm study, or if it was a retrospective study, case report, editorial, letter, commentary, or review article. Two independent reviewers reviewed and extracted the data from eligible studies. If there was a disagreement between the 2 reviewers, a third reviewer resolved the issue.

### Data extraction

The following information was extracted from the included studies: study type, number of patients who received intravenous or intra-arterial thrombolysis, age, gender, and baseline National Institutes of Health Stroke Scale (NIHSS) score. We extracted data on the following clinical outcomes resulting from therapy: the proportion of patients that showed treatment benefit, experienced neurological deterioration or intracranial hemorrhage, required recanalization, or died.

### Statistical analysis

The clinical outcome measures analyzed in the meta-analysis were modified Rankin scale (mRS) score and mortality, both at 90 days. A favorable outcome was defined as an mRS score of 0 to 2. The odds ratio (OR) and 95% confidence interval (CI) were calculated for binary outcomes and compared between patients who received IAT or IVT. For the mRS score, a favorable outcome, an OR > 1, indicates that the IAT group is favored; in contrast, for mortality, an OR >1 indicates that the IVT group is favored. A χ2-based test of homogeneity was performed, and the inconsistency index (I^2^) statistic was determined. If I^2^ was > 50% or > 75%, then the trials were considered to be heterogeneous or highly heterogeneous, respectively. An I^2^ < 25% indicated homogeneity among the studies. When heterogeneity existed between studies (I^2^ > 50%), a random-effects model was used. Otherwise, fixed-effects models were used. Pooled summary statistics for ORs of the individual studies were reported. Sensitivity analysis was performed by using the leave-one-out approach. Publication bias was not assessed because more than 5 studies are required to detect funnel plot asymmetry [27]. All analyses were performed by using Comprehensive Meta-Analysis statistical software, version 2.0 (Biostat, Englewood, NJ). A value of *P* < 0.05 was considered to indicate statistical significance. In addition, meta-regression was used to determine if differences in baseline characteristics affected the pooled estimates by using SPSS software version 17 (SPSS Inc, Chicago, IL) [28].

## Results

### Basic characteristics and systematic review of studies

**Figure 1:**
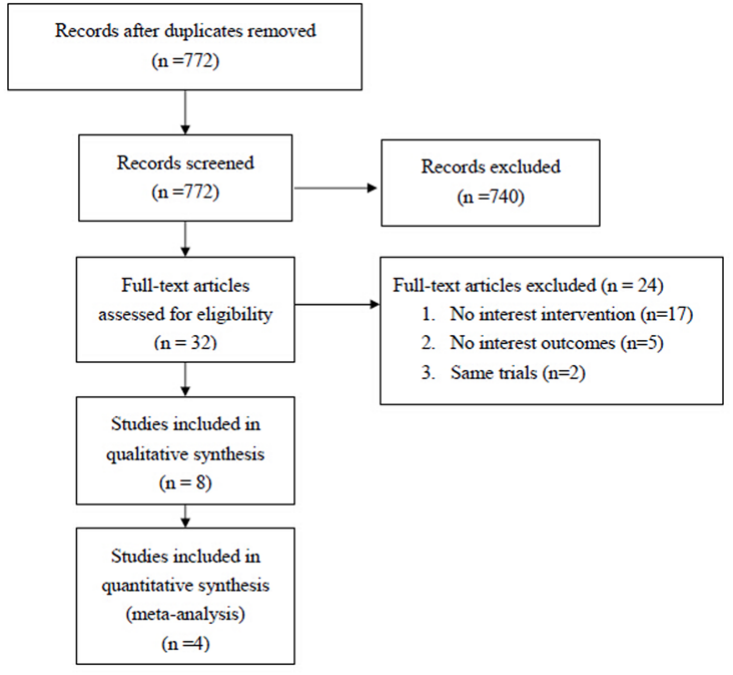
A flow diagram for study selection.

The database search identified 772 studies, of which 740 did not meet the inclusion criteria (Fig. 1). Thirty-two studies were reviewed in detail and 24 were excluded because they did not investigate IAT or IVT (n = 17), did not report relevant treatment outcomes (n = 5), or were a secondary report of an included study (n = 2). Eight studies [13, 18–20, 24–26, 29], representing 855 patients were included in the systematic review and 4 of the 8 were used in the meta-analysis [18, 25, 26, 29]. Four studies were excluded from the meta-analysis because of pooled results across multiple types of treatment [24], lack of numerical information on clinical outcomes [20], no use of a thrombolytic in IVT [13], or use of a combination of IAT and IVT [19].

Six of the 8 studies were randomized control trials (Table 1) [13, 19, 20, 24–26]. Overall, studies were similar with respect to the distributions of age and gender among patients in the IAT and IVT groups. The mean age ranged from 60 to 66 years for the IAT group and from 58 to 68 years for the IVT group, and the proportion of men was 53% to 92% in the IAT group and 56% to 79% in the IVT group (Table 1). The NIHSS score at baseline ranged from 9 to 17 for both groups. The inclusion criteria of 4 of the studies used the NIHSS: 1 study required a score between 4 and 24, 2 a score ≥4, and 1 a score ≥5. One study required a score of ≤50 on the Scandinavian Stroke Scale, while the inclusion criteria of 3 studies did not require administration of a stroke scale (Table 1).

**Table 1:** Summary of characteristics of included studies.

**First Author (Year)**	**Study type**	**Inclusion criteria**	**Comparison**		**Number of cases**	**Age (years)**	**Men, n**	**Median baseline NIHSS score**	**Time from stroke onset to treatment**
			**IAT**	**IVT**			**(%)**		
Ciccone A (2013)	RCT	Age 18–80; Stroke onset to initiation of IVT was defined as within 4.5 hours or for the administration of IAT treatment within 6 hours.	Endovascular treatment ^§^	tPA	181 vs. 181	66 vs. 67	59 vs. 57	13 vs. 13	Median 225 (194–260) vs. 165 (140–200) mins
Ciccone A (2010)	RCT	Age 18–80; Stroke onset to initiation of IVT was defined as within 3 hours or for the administration of IAT treatment within 6 hours.	Alteplase	Alteplase	25 vs. 29	61 vs. 64	76 vs.79	17 vs. 16	Median 195 (170–240) vs. 155 (135–170) mins
Zhang B (2010)	Prospective	Age 18–80; NIHSS score 4–24;	tPA	Alteplase	23 vs. 55	62 vs. 67	78 vs. 75	9 vs. 9	Mean 4.6 ± 1.3 vs. 3.1 ± 0.5 hrs
Sen S (2009)	RCT	Age>18; NIHSS≧4 or isolated aphasia or isolated hemianopsia; Time of presentation <3 h from symptom onset.	tPA	tPA	3 vs. 4	All: 68	All: 71	All: 16	Mean 120 ± 42 vs. 95 ± 35 mins ^a^
Mattle HP (2008)	Prospective	Treated with IVT in the 3-hour window. Treated with IAT in the 6-hour window;	Urokinase	tPA	55 vs. 57	61 vs. 61	61 vs. 67	Mean: 17.5 vs. 16.7	Mean 244 ± 63 vs. 156 ± 21 mins
Ducrocq X (2005)	RCT	Age 18–79; Scandinavian Stroke Scale score ≦50; Onset to treatment within 6 hours.	Urokinase	Urokinase	13 vs. 14	60 vs. 58	92 vs. 64	NA	Mean 5.24 vs. 4.16 hrs
Furlan A (1999)	RCT	Age 18–85; NIHSS score≧4 or isolated aphasia or isolated hemianopsia; Onset to treatment within 6 hours.	Prourokinase (IAT)+ heparin (IVT)	Heparin (IVT)	121 vs. 59	64 vs. 64	58 vs. 61	17 vs. 17	Median 4.7 (4.0–5.3) vs. 5.1 (4.2–5.5) hrs ^b^
Lewandowski CA (1999)	RCT	Age≦84; NIHSS score>5	tPA (IVT+IAT)	tPA (IAT)	17 vs. 18	66 vs. 67	53 vs. 56	16 vs. 11	Median 2.6 (2.3–2.8) vs. 2.7 (1.9–2.9) hrs

RCT: randomized controlled trial; NA: not available; IAT: intra-arterial thrombolysis; IVT: intravenous thrombolysis; tPA: tissue plasminogen activator; NIHSS: National Institutes of Health Stroke Scale; mins, minutes; hrs, hours;

^§^, endovascular treatment included IAT of tPA, mechanical thrombolysis or both;

^a^, time from arrival to initiation of thrombolysis;

^b^, time from stroke onset to randomization

The studies showed variability not only in the requirement of a stroke scale score, but also in the maximum amount of time that should elapse between the stroke onset and the time to treatment. The inclusion criteria of 2 studies did not limit enrollment on the basis of time since stroke onset, 2 studies required patients of both groups to be enrolled within 6 hours, 2 studies required enrollment of patients into the IVT group within 3 hours and the IAT group within 6 hours, 1 study required enrollment of patients into the IVT group within 4.5 hours and the IAT group within 6 hours, and 1 study required that all enrolled patients must have presented for treatment within 3 hours of stroke onset (Table 1). Due to these differences in enrollment criteria, the time that elapsed from stroke onset to treatment for the 5 studies that reported data ranged from approximately 2.3 to 5.9 hours for the IAT group and 1.9 to 3.6 hours in the IVT group (Table 1).

**Figure 2:**
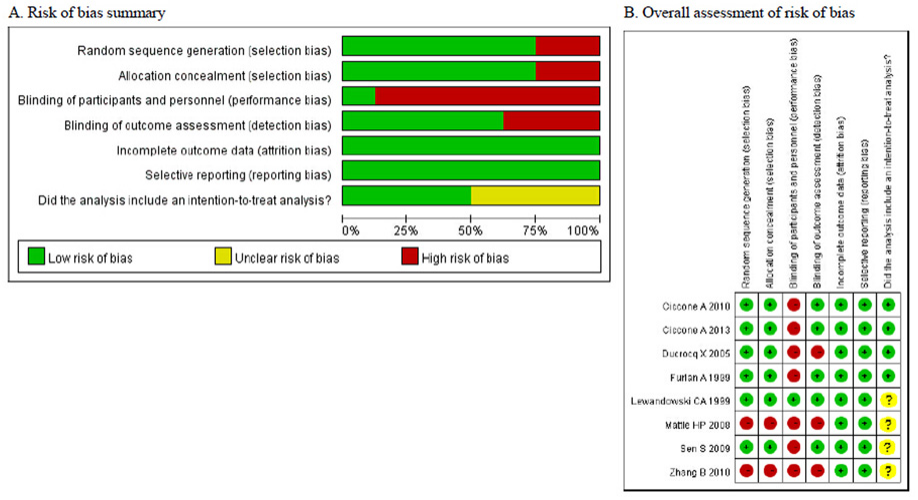
Assessment of the risk of bias among the included studies.

Assessment of the risk of bias indicated that 7 of the 8 studies had performance bias related to blinding of either the subjects or study personnel (Fig. 2). Five of these were open label studies [13, 20, 24–26]. An intent-to-treat analysis was only used in 4 studies [13, 24–26]. The studies of Mattle et al (2008) and Zhang et al (2010) were also biased in regard to randomization, selection, and blinding of outcome assessments [18, 29], all of which indicates that the data were of lower quality.

### Systematic review

Three of the 8 studies determined that patients with acute ischemic stroke received similar benefit from IAT and IVT with respect to favorable outcome, mortality, and safety [24–26]. In a randomized, open-label, controlled study, Ciccone et al (2013) evaluated survival-free disability (defined as an mRS score of 0 to 1) in patients treated with either IVT (n = 181) or IAT (n = 181) in conjunction with recombinant tissue plasminogen activator (tPA) [24]. At 3 months, 30.4% of patients in the IVT group and 34.8% of patients in the IAT group were alive without disability (OR, 0.71; 95%CI, 0.44 to 1.44; *P* = 0.16), and 55% and 54% of patients, respectively, had an NIHSS score ≤6 (Table 2) [24]. They also found that 7% and 9% of patients, respectively, had neurological deterioration: symptomatic cranial hemorrhage occurred in 6% of patients in each group, with 4 patients in the IVT group and 1 in the IAT group dying from hemorrhage (Table 2). Overall, 8% of IVT and 6% of IAT patients died during the study. There was no significant difference between groups in the incidence of other serious adverse events [24].

**Table 2:** Clinical outcomes of the included studies.

**First Author (Year)**	**Comparison**	**Favorable outcome, ^a^ %**	**Neurological deterioration, ^b^ %**	**Recanalization (TIMI 2+3), %**	**Intracranial hemorrhage, %**	**Mortality, %**
Ciccone A (2013)	IAT vs. IVT	30.4 vs. 34.8	9 vs. 7	NA	6 vs. 6 (7 d) ^§^	8 vs. 6 (day 7)
						14.4 vs. 9.9 (day 90)
Ciccone A (2010)	IAT vs. IVT	56 vs. 31	0 vs. 4	NA	8 vs. 14 (7 d) ^§^	20 vs. 14 (day 7)
Zhang B (2010)	IAT vs. IVT	82.6 vs. 56.4 *	NA	82.6 vs. NA	8.7 vs. 9.1 ^§^	8.7 vs. 16.4 (day 90)
					30.4 vs. 12.7	
Sen S (2009)	IAT vs. IVT	NA	NA	100 vs. 0 *	0 vs. 25 (24 hrs) ^§^	NA
					33 vs. 25 (24 hrs)	
Mattle HP (2008)	IAT vs. IVT	53 vs. 23 *	NA	71 vs. NA	1.8 vs. 0	7 vs. 23 (day 90)*
Ducrocq X (2005)	IAT vs. IVT	46 vs. 29	NA	All: 15	15 vs. 0 ^§^	23 vs. 29 (day 90)
Furlan A (1999)	IAT vs. IVT	40 vs. 25 *	NA	66 vs. 18 (2hr)*	10 vs. 2 (24 hrs)	25 vs. 27 (day 90)
Lewandowski CA (1999)	IAT vs. IVT	47 vs. 67	NA	82 vs. 50 (2hr)	0 vs. 5.5 (24 hrs) ^§^	29 vs. 5.5 (day 90)
					11.8 vs. 5.5 (72 hrs) ^§^	

NA: not available; IAT: intra-arterial thrombolysis; IVT: intravenous thrombolysis; tPA: tissue plasminogen activator; TIMI: Thrombolysis In Myocardial Infarction

^a^Favorable outcome was defined as a modified Rankin Scale score of 0 to 2 at 90 days after stroke.

^b^Neurological deterioration was defined as an NIHSS score ≥ 4 points on day 7.

^*^, *P* value < 0.05. §, symptomatic intracranial hemorrhage.

^§^, symptomatic intracranial hemorrhage.

In another randomized controlled study, Ciccone et al (2010) compared the efficacy and safety of either IAT or IVT and Alteplase for treatment of acute ischemic stroke [25]. Although there were twice as many patients in the IAT group (n = 25) compared with the IVT group (n = 29) who survived to 90 days without residual disability (12/25 vs. 8/29), the difference did not reach statistical significance (OR, 3.2; 95%CI, 0.9 to 11.4; *P* = 0.67). At day 7, a similar proportion of patients in both treatment groups had experienced symptomatic intracranial hemorrhage (*P* = 0.675) and had died (*P* = 0.718) (Table 2). There was no difference in the rate of serious adverse events between treatment arms [25]. Similarly, another randomized study by Ducrocq et al (2005) did not find a significant difference between either IAT or IVT in conjunction with urokinase therapy for favorable outcome (mRS score ≤2 (*P* = 0.06), symptomatic hemorrhage (*P* = 0.4), or mortality (*P* = 0.90) (Table 2) [26]. However, the study was small (N = 27) and was prematurely terminated owing to mortality. Accordingly, the findings are difficult to interpret.

In contrast, 4 studies found a benefit of IAT compared with IVT in treating patients with acute ischemic stroke [13, 18, 20, 29]. Mattle et al (2008) found more benefit from therapy with IAT in a non-randomized prospective study that compared IAT with urokinase to IVT with tPA for the treatment of stroke patients with hyperdense middle cerebral artery sign (HMCAS) (N = 112) [18]. A greater proportion of patients had a favorable outcome (mRS score 0 to 2) after IAT (53%) compared with IVT (23%; *P* = 0.001), and mortality was lower with IAT than with IVT (7% vs. 23%; *P* = 0.022). The rate of symptomatic intracranial hemorrhage was similar between treatment groups (*P* = 0.16), and recanalization occurred in 71% of IAT-treated patients (Table 2) (recanalization was not systematically assessed in IVT-treated patients so no data were presented) [18]. The study by Zhang et al (2010), also a prospective study, enrolled 78 patients who were treated with either IAT or IVT [29]. These authors observed that patients who received IAT were more likely to have a favorable outcome than were patients in the IVT group (*P*= 0.028, RR = 2.66, 95%CI:1.10 to 7.04). The 2 groups of patients experienced intracranial hemorrhage (any type) at rates that were not significantly different (*P*= 0.103, RR = 2.391, 95%CI: 0.946 to 6.047); the rates of symptomatic intracranial hemorrhage and mortality were also similar (*P*= 1.00, RR = 0.957, 95%CI: 0.200 to 4.579 and *P*= 0.492, RR = 1.882, 95%CI: 0.440 to 8.045, respectively). This study did assess recanalization of patients in the IAT group and reported a rate of 82.6% (Table 2).

In a randomized controlled study (N = 7) that compared IAT to IVT with recombinant tPA for treatment of patients with acute ischemic stroke with major vessel inclusion, Sen et al (2009) also found that IAT showed a trend toward a higher recanalization rate (3/3 patients for IAT and 0/4 patients for IVT; *P* = 0.03) (Table 2) [20]. They found no difference in the frequency of symptomatic hemorrhage or neurological improvement (*P* = 1.0) [20]. A major limitation of this study was the small study population [20]. Furlan et al (1999) conducted a randomized controlled trial (N = 180) that compared IAT with prourokinase (n = 121) to IVT with no thrombolytic agent (n = 59) [13]. They found that favorable outcomes were more numerous among patients who received IAT than among those who received IVT (40% vs. 25%; *P* = 0.04) at 90 days (Table 2) [13]. The recanalization rate was 66% for the IAT group and 18% for the IVT group (*P* < 0.001). However, symptomatic intracranial hemorrhage within 24 hours was greater in the IAT group than in the IVT group (10% vs. 2%; *P* = 0.06).

A randomized controlled Phase I study by Lewandowski et al (1999) (N = 35) evaluated the efficacy and safety of both IAT with tPA and the combination of IVT and IAT with tPA [19]. There was no difference in favorable outcome at 90 days (mRS score 0–1) between treatments (*P* = 0.20); however, 35% of IAT patients had a favorable outcome regardless of IVT treatment [19]. Recanalization was more frequent in the IVT/IAT combined therapy group than in the IAT treatment group (*P* = 0.03) (Table 2) [19]. One patient in the IAT group and 2 in the IVT/IAT group had a symptomatic intracranial hemorrhage in the first 24 hours [19]. Although a greater proportion of patients died in the IVT/IAT group than in the IAT group (29% vs. 5.5%, respectively), the difference did not reach statistical significance (*P* = 0.06) [19].

### Meta-analysis of outcome measures

**Figure 3:**
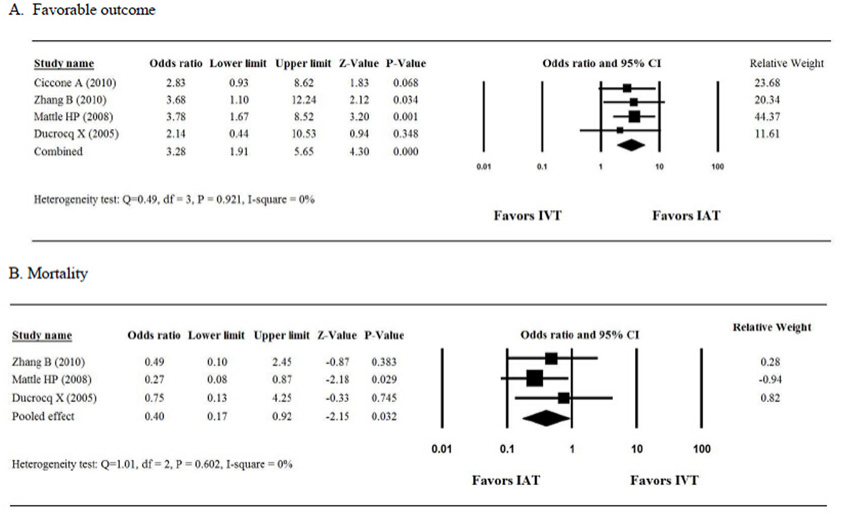
Forest plots showing the results of the meta-analyses.

The 4 studies that were analyzed for favorable outcome [18, 25, 26, 29] included 116 patients who were treated with IAT and 155 who were treated with IVT. The ORs for favorable outcome for the 4 studies ranged from 2.14 to 3.78 (Fig. 3A). We detected homogeneity in the combined OR of the 4 studies (Q = 0.49, I^2^ = 0%, *P* = 0.921); therefore, a fixed-effects model of analysis was used. The combined OR of 3.28 (95%CI, 1.91 to 5.65; *P* < 0.001) indicates that patients who received IAT had a significantly greater chance of a favorable outcome than did those who received IVT.

The study by Ciccone et al (2010) was excluded from the meta-analysis of mortality because it reported results at 7 but not at 90 days [25]. As a result, we analyzed the mortality rate among 3 studies that enrolled a total of 91 and 126 patients in the IAT and IVT groups, respectively. The ORs for mortality ranged from 0.27 to 0.75 (Fig. 3B). When the data were pooled for analysis, we determined that the 3 studies were homogenous (Q = 1.01, I^2^ = 0%, *P* = 0.602); therefore, a fixed-effects model of analysis was used. Examination of the combined OR revealed a significant difference between patients who received IAT and those who received IVT (combined OR 0.40; 95%CI, 0.17 to 0.92; *P* = 0.032. This finding indicates that IAT therapy significantly reduced the proportion of patients who had died by 90 days.

**Figure 4:**
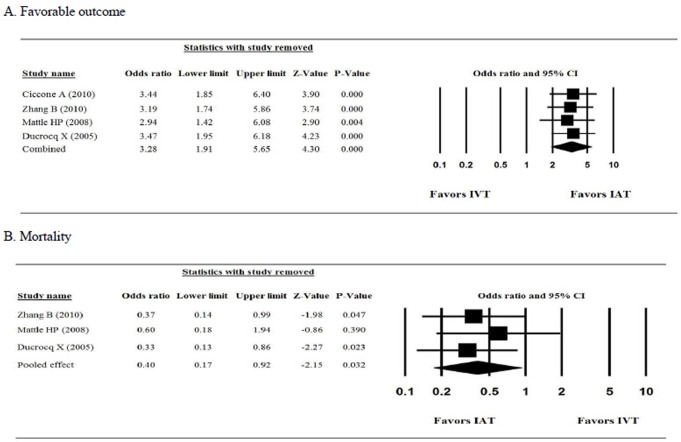
The results of sensitivity analysis. (A) Favorable outcome, (B) Mortality.

Sensitivity analysis of the mRS score and mortality with 1 study removed in turn indicated that the direction and magnitude of the combined estimates did not change. The ORs showed little variation across the analysis, indicating that no one study dominated the pooled estimates (Fig. 4). We did not assess the publication bias of the studies because standard analytical techniques (Funnel plot, Egger’s test) are not recommended if less than 5 studies are being analyzed [27].

### Meta-regression of favorable outcome

After adjusting for baseline covariates (age, NIHSS score, and time to treatment initiation), we determined that patients who received IAT still had a significantly greater chance of a favorable outcome than did those who received IVT (OR: 1.98, *P*= 0.036).

## Discussion

Intra-arterial thrombolysis has been advocated for treatment of acute ischemic stroke. This meta-analysis evaluated the efficacy of IAT compared with IVT in treating this condition. We found the IAT was associated with a significantly greater favorable outcome, as evaluated with the mRS score, compared with IVT (*P*<0.001). There was also a significant difference between treatments in the rates of mortality.

Our findings differ from prior meta-analyses. A study by Mullen et al (2012) compared the relative efficacy of 6 reperfusion strategies: intravenous with Alteplase; intra-arterial Alteplase, Urokinase, or Reteplase; intra-arterial mechanical thrombolysis; mechanical thrombolysis and intra-arterial Alteplase, Urokinase, or Reteplase; and 1 of 2 doses of intravenous Alteplase followed by intra-arterial chemical and intra-arterial mechanical thrombolysis, either alone or in combination [30]. This analysis included 54 studies that enrolled a total of 5019 subjects. The included studies consisted of retrospective (n = 20) and prospective (n = 27) studies, as well as of studies that could not be categorized (n = 7). The majority of patients received intravenous tPA (n = 2450), intra-arterial chemical thrombolysis (n = 1143), or the combination of intra-arterial mechanical and chemical thrombolysis (n = 819). The meta-analysis found a significant difference across treatments in mean mRS score (*P*< 0.0001) and mortality (*P*= 0.0024). However, in a meta-regression analysis that controlled for baseline covariates including baseline NIHSS score, time to treatment initiation, treatment type, and other baseline characteristics, they found no difference in mRS score among reperfusion treatments. The authors concluded that differences across treatments reflect baseline characteristics and not treatment effect [30]. The difference between the findings of Mullen et al (2012) and our own study may reflect a difference in study inclusion criteria. For example, their analysis included a wide range of study types such as case reports and observational studies, while ours only included randomized controlled prospective studies.

Two other meta-analyses also found no benefit of IAT over IVT [31, 32]. A meta-analysis by Wardlaw et al (2013) that included 2527 participants from 20 unconfounded randomized or quasi-randomized trials examined the differences among clot-dissolving drugs, dose ranges of the same agent, and IAT and IVT [31]. The pooled estimate of 5 studies that compared IAT and IVT points to no benefit of IVT over IAT, in regard to mortality (combined OR, 0.81; 95%CI, 0.47 to 1.39) or symptomatic intracranial hemorrhages (OR, 1.10; 95%CI, 0.4 to 2.25). This prior analysis did not evaluate the mRS score, so we were not able to directly compare our findings with theirs. The second meta-analysis, that of Nam and colleagues (2013) [32], analyzed 4 trials that enrolled 351 patients. The clinical endpoints that were studied included functional outcome (good or excellent), mortality, incidence of intracranial hemorrhage, and intracranial hemorrhaging that resulted in death. Among the 2 studies that defined a good functional outcome as an mRS score of 0 to 2, the pooled effect for a favorable outcome tended to favor IAT over IVT (poor outcome: risk ratio (RR), 0.68; 95%CI, 046 to 1.00; *P*= 0.05), but found no clear benefit of either approach in regards to mortality (RR, 1.12; 95%CI, 0.47 to 2.68; *P*= 0.79). These 2 studies assessed by Nam et al (Ciccone et al, 2010 and Ducrocq et al, 2005), which were also included in our meta-analysis, enrolled only 81 patients and analyzed data for mortality at substantially different time points (7 and 90 days). For this reason, we believe our results are more robust.

The recanalization rate following IAT or thrombolytics with or without mechanical treatments is about 60% to 80%, and reocclusion after successful thrombolysis is approximately 22% to 34% [33]. A variety of combination therapies have been tested for their ability to improve clinical outcomes. One randomized study by Qureshi et al (2006) of patients with acute ischemic stroke (3–6 hours after symptom onset) found that the combination of intra-arterial Reteplase in conjunction with intravenous Abciximab was safe and resulted in partial or complete recanalization in 13 of 20 patients [34]. Another study by Kim et al (2012) evaluated the feasibility and safety of intra-arterial Tirofiban after formation of anterograde flow following mechanical thrombolysis in acute ischemic stroke [35]. They found that approximately 50% of patients showed clinical improvement and had a favorable outcome at 24 hours and 3 months post treatment. These findings indicate that this treatment is a viable option for patients with acute ischemic stroke [35]. One study investigated the use of intra-arterial Eptifibatide in patients who had reocclusion and who were receiving endovascular treatment for ischemic stroke. These authors determined that intra-arterial Eptifibatide was efficacious for salvage of reocclusion.

Our analysis has several limitations that should be considered when interpreting our findings. We included only a small number of studies, and the small patient population in Sen et al may increase bias in the analysis. In addition, the studies included in this meta-analysis did not use the same mechanical devices and used variable doses of heparin and the other drugs, which were not administered the same way, and different thrombolytic agents and intra-arterial techniques. All of these factors may have influenced the resulting outcomes. However, other studies have taken a similar approach to conducting meta-analyses; therefore, this may be a limitation that is common to studies such as our own.

In conclusion, this meta-analysis determined that IAT conferred a significantly greater chance of achieving a favorable outcome compared with IVT. There was also a difference between IAT and IVT for the risk of mortality. The studies included in this analysis were small and heterogeneous, and larger randomized clinical studies are necessary to further investigate this issue.

## Supporting Information

S1 Checklist PRISMA Checklist. (DOC)

S1 Diagram PRISMA flow diagram. (DOC)
