# Supplementary material for: Intravenous versus Intra-Arterial Thrombolysis in Ischemic Stroke: A Systematic Review and Meta-Analysis
Source: PLoS One. 2015 Jan 8;10(1):e0116120. doi: 10.1371/journal.pone.0116120 (PMC4287629; doi:10.1371/journal.pone.0116120)
Supplement: S1 Diagram — (DOC) [file pone.0116120.s002.doc]

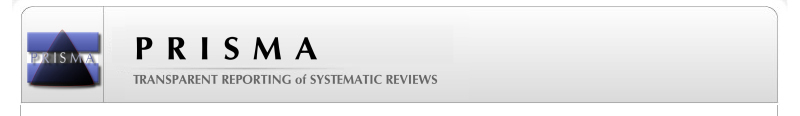
**PRISMA 2009 Flow Diagram**

**Screening**

**Included**

**Eligibility**

**Identification**

Records identified through database searching
(n = 771 )

Additional records identified through other sources
(n = 0 )

Records after duplicates removed
(n = 771 )

Records screened
(n =771 )

Records excluded
(n =740 )

Full-text articles assessed for eligibility
(n = 31 )

Full-text articles excluded, with reasons(n =24 )

1. No interest intervention (n=17)
2. No interest outcome (n=5)
3. Same trials (n=2)

Studies included in qualitative synthesis
(n = 7 )

Studies included in quantitative synthesis (meta-analysis)
(n = 4 )
